# A machine learning approach to radiogenomics of breast cancer: a study of 922 subjects and 529 DCE-MRI features

**DOI:** 10.1038/s41416-018-0185-8

**Published:** 2018-07-23

**Authors:** Ashirbani Saha, Michael R. Harowicz, Lars J. Grimm, Connie E. Kim, Sujata V. Ghate, Ruth Walsh, Maciej A. Mazurowski

**Affiliations:** 1 Department of Radiology, Duke University School of Medicine, Durham, NC 22705 USA; 2 Department of Electrical and Computer Engineering, Duke University, Durham, NC 22705 USA; 3 Duke University Medical Physics Program, Durham, NC 22705 USA

**Keywords:** Breast cancer, Predictive markers

## Abstract

**Background:**

Recent studies showed preliminary data on associations of MRI-based imaging phenotypes of breast tumours with breast cancer molecular, genomic, and related characteristics. In this study, we present a comprehensive analysis of this relationship.

**Methods:**

We analysed a set of 922 patients with invasive breast cancer and pre-operative MRI. The MRIs were analysed by a computer algorithm to extract 529 features of the tumour and the surrounding tissue. Machine-learning-based models based on the imaging features were trained using a portion of the data (461 patients) to predict the following molecular, genomic, and proliferation characteristics: tumour surrogate molecular subtype, oestrogen receptor, progesterone receptor and human epidermal growth factor status, as well as a tumour proliferation marker (Ki-67). Trained models were evaluated on the set of the remaining 461 patients.

**Results:**

Multivariate models were predictive of Luminal A subtype with AUC = 0.697 (95% CI: 0.647–0.746, *p* < .0001), triple negative breast cancer with AUC = 0.654 (95% CI: 0.589–0.727, *p* < .0001), ER status with AUC = 0.649 (95% CI: 0.591–0.705, *p* < .001), and PR status with AUC = 0.622 (95% CI: 0.569–0.674, *p* < .0001). Associations between individual features and subtypes we also found.

**Conclusions:**

There is a moderate association between tumour molecular biomarkers and algorithmically assessed imaging features.

## Introduction

Radiogenomic^1^ (a.k.a. imaging-genomic) analysis of breast cancer, which investigates the relationship between breast tumour imaging characteristics and tumour molecular, genomic, proliferation, and related features, has been gaining significant interest in recent years.^2–18^ Establishing a strong relationship between tumour imaging phenotypes and molecular markers could provide a non-invasive surrogate means of genomic analysis. This could be done by using a non-invasive imaging signature instead of a genomic signature that requires invasive tissue sampling. Otherwise, these relationships could help identify groups of patients that may benefit from additional genomic analysis.

Previous studies^2,3,5,7–16,18^ have demonstrated the potential for radiogenomic associations in breast cancer, predominantly associations of MR imaging features with molecular subtype or gene assays to predict cancer recurrence. However, as shown in Table 1, the majority of the prior studies on breast radiogenomics have used moderate sample sizes (most studies to date used <100 subjects) and small numbers of imaging features (typically <100). Also, only two prior studies have used an independent validation set to study the predictive ability of their imaging features.^3,18^ Consequently, the reported strengths of the imaging associations have varied widely. For example, for discriminating triple-negative breast cancer versus other subtypes, area under the curve (AUC) values of 0.92 in a study,^3^ 0.79 in another study,^18^ 0.78 in a different study,^11^ and 0.67 in another study^17^ were reported. Furthermore, each study uses a different, often very limited, set of imaging features which renders the comparison of specific results infeasible.

**Table 1:** Prior studies reporting association of breast MR imaging features and genomic characteristics

First author, year and reference	Number of imaging features from MR	Number of patients (dataset information: S for single and M—for multiple institutions)	Breast cancer related principal research question
Uematsu et al.^9^	9	176 (S)	Correlation of Imaging features and pathologic findings in TNBC and non-TNBC
Costantini et al.^14^	14	225 (S)	Comparison of imaging features of TNBC and non-TNBC
Yamamoto et al.^7^	26	10 (M)	Association of imaging features and interferon breast cancer subtype
Sung et al.^8^	7	321 (S)	Comparison of imaging features of TNBC and non-TNBC
Agner et al.^13^	120	65 (S)	Imaging features for predicting TNBC and other cancers
Mazurowski et al.^15^	23	48 (M)	Association of imaging features and molecular subtypes
Blaschke et al.^10^	6	112 (S)	Association of imaging features and molecular subtypes
Grimm et al.^6^	56	275 (S)	Association of imaging features and molecular subtypes
Guo et al.^12^	38	91 (M)	Integrated radiomics and genomics data to predict clinical phenotypes
Wang et al.^11^	85	84 (M)	Association of imaging features of background parenchymal enhancement and TNBC
Yamaguchi et al.^16^	5	186 (S)	Association of imaging features and molecular subtypes
Li et al.^17^	37	91 (M)	Association of imaging features and molecular subtypes
Fan et al.^3^	88	96 (S)	Association of imaging features and molecular subtypes
Wu et al.^18^	35	210 (M)	Association of imaging features and molecular subtypes

TNBC triple negative breast cancer

In this study, we address this issue by presenting a comprehensive study of associations of tumour surrogate molecular subtype, receptor status, and proliferation of a set of 922 patients and 529 MR imaging features. The dataset’s heterogeneous imaging parameters (manufacturer, magnetic field strength, acquisition parameters) ensure that the results are not limited to a very specific MR setting. The set of features used in this study was constructed to represent a wide range of imaging characteristics including size, shape, texture, and enhancement of both the tumour and the surrounding tissue. We included both features published in the literature as well as those developed in our laboratory and developed machine learning-based multivariate models to test the effectiveness of these features.

## Materials and methods

### Patient population

**Fig. 1:**
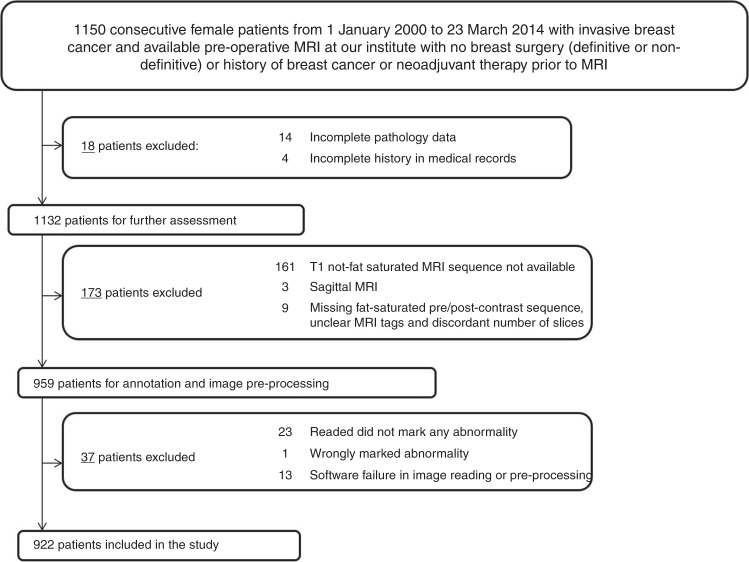
Flowchart of inclusion and exclusion criteria for patients

In this local institutional board approved study, we identified 1150 consecutive female patients, from 1 January 2000 to 23 March 2014 with invasive breast cancer and available pre-operative MRI at our institution. A waiver for informed consent was also secured for this study. Patients with prior breast surgery, history of breast cancer, or neoadjuvant therapy prior to the MRI acquisition were excluded. From these patients, 922 patients were selected for our study using the specific criteria shown in Fig. 1. These 922 patients included 271 patients that were used in our earlier much more limited analyses^6,19^ which in addition to analysing a notably smaller cohort, did not investigate the associations of MR imaging features with ER, PR, and HER2 status, and proliferation marker (Ki-67). Our previous studies with the subset of this cohort also did not validate the findings on an independent test set.

### Pathology data

Pathology results from the first immunohistochemistry (IHC) analysis, or the clinician’s note if not available (*n* = 65), were reviewed for the ER, PR, and H2 status. An Allred score from the IHC greater than or equal to 3+ was considered positive for ER and PR. For the determination of HER2 status, an IHC HER2 score of 3+, or a score of 2+ with an additional condition of amplification of HER2 gene by FISH (PathVysion Her2 DNA Probe kit, Abbott Laboratories, Chicago, IL)^6^ was considered positive. Following the criteria described in earlier publications,^20,21^ the surrogate molecular subtype was determined as: Luminal A (ER and/or PR positive, HER2 negative), Luminal B (ER and/or PR positive, HER2 positive), HER2 (ER and PR negative, HER2 positive), triple-negative (ER, PR, and HER2 negative). We also recorded the proliferation marker (Ki-67) values from the initial IHC data, when available. Ki-67 was considered high if it was greater than 14 as in refs.^22,23^. Additionally, the majority (84%) of tumours were ductal, 10% were lobular, 4% belonged to other categories, and 4% did not have this data available.

### Imaging data

For the patients included in our study, we collected axial breast MRIs that were acquired by 1.5T or 3T scanners in the prone position. Scanner related details and MR acquisition parameters can be found in Supplementary material (Tables S1 and S2, respectively). The following MRI sequences were available: a non-fat saturated T1-weighted sequence, a fat-saturated gradient echo T1-weighted pre-contrast sequence, and typically four post-contrast T1-weighted sequences acquired after the IV administration of contrast agent (using a weight-based protocol of 0.2 mL/kg). In our cohort, three types of contrast agents were used as follows: gadobutrol (Gadavist, Bayer Healthcare, Berlin, Germany) for 2 (0.2%) patients, gadopentetate dimeglumine (Magnevist, Bayer Healthcare, Berlin, Germany) for 560 (60.8%) patients, and gadobenate dimeglumine (Multihance, Bracco, Milan, Italy) for 263 (28.5%) patients. The specific name of the contrast agent used was not available for 97 (10.5%) patients. The median acquisition time between a pair of post-contrast sequences was 131 s.

Each case was annotated by one of eight fellowship-trained breast imagers (1–22 years of post-fellowship experience, 3–32% of cases annotated by different readers). For each patient, a graphical user interface developed in our laboratory displayed to the reader the following MR sequences: (a) pre-contrast, (b) first post-contrast, and (c) subtracted (obtained by subtracting the pre-contrast from the first post-contrast). Tumours were delineated by three-dimensional boxes provided by the reader.

### Image segmentation

Using a reader’s annotation (3D box), we applied a fuzzy C-means automatic segmentation^24^ to obtain the tumour mask. The breast and fibroglandular tissue masks were automatically extracted from the N4-corrected^25^ T1-non-fat saturated (T1-NFS) images and first post-contrast sequences. Thus, we had four masks extracted for each patient: (a) tumour (semi-automatic) (b) breast mask (automatic) (c) fibroglandular tissue (FGT) mask from T1-NFS (automatic) (d) FGT mask from post-contrast sequence (automatic). We removed any tumour voxels overlapping with the FGT masks to arrive at the final FGT masks used for feature extraction.

### Imaging feature extraction and organisation

**Fig. 2:**
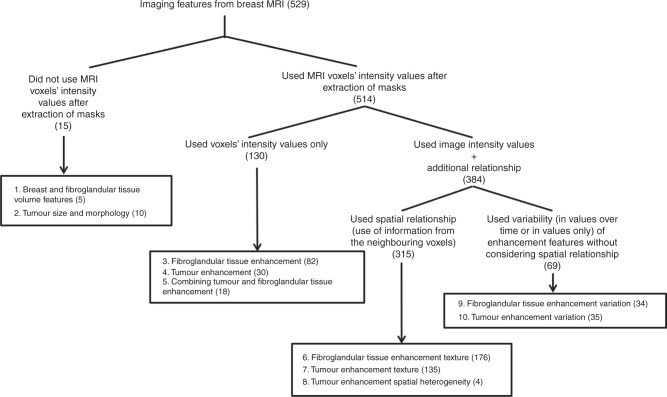
Feature distribution as per the different groups

A review of the literature on breast MR image processing, computer-aided diagnosis, and radiomics guided our feature selection. The goal was to compile a comprehensive set of features that have been shown to be effective predictors and could quantify characteristics of the breast, tumour, and FGT. Based on the source and the nature of image processing represented by a feature, we categorised all features as shown in Fig. 2. Based on the type of the feature extracted, different subsets of the available MR sequences were used (all of them if necessary).

Features were derived from the following sources: (a) features that capture the properties of breast as a whole are in category 1, (b) categories 2, 4, 7, 8 and 10 quantify characteristics of the tumour, (c) characteristics of FGT are expressed by features included in categories 1, 3, 6 and 9, and (d) category 5 represents features that capture the properties of tumour and FGT enhancement simultaneously. As per the image processing, (a) features in categories 1 and 2 do not require the voxel intensity values after the extraction of the corresponding masks (b) features in categories 3, 4 and 5 use voxel intensity values for capturing tumour/tissue enhancement but the spatial relationship of the voxels are not explored, (c) features in categories 6, 7 and 8 exploit the spatial relationship of the voxels while quantifying enhancement (d) features in categories 9 and 10 use variation in enhancement over time or in values but does not use the spatial relationship.

The list of the features are available in the Appendix (Supplementary Material A) of our previous publication.^26^

### Training and test sets

Half (461) of the available 922 patients were included in our training set and the remaining 461 were included in our test set. The training set included 271 patients that were used in our earlier analyses^6^ to minimise the potential bias that could arise by including such cases in the test set since we have worked with this subset of data previously. A random split of the remaining data was used to arrive at the equal split between the training set and the test set.

### Model development and statistical analysis

The primary goal of this study was to evaluate whether machine learning-based multivariate models based on imaging features can distinguish between different tumour subtypes and can predict molecular, genomic, and proliferation markers such as ER status, PR status, HER2 status, and proliferation (Ki-67). We considered eight-specific prediction tasks corresponding to eight radiogenomics associations where an imaging-based model aims to distinguish: (1) Luminal A versus other subtypes, (2) Luminal B versus other subtypes, (3) HER2 versus other subtypes, (4) TNBC versus other subtypes, (5) ER positivity versus OR negativity, (6) PR positivity versus PR negativity, (7) HER2 positivity versus HER2 negativity, and (8) high Ki-67 versus low Ki-67.

A multivariate model for each of these 8 tasks was developed using the training set in a following manner. First, *N* features with the highest area under the receiver operating characteristic (ROC) curve (AUC) were selected using the corresponding feature as the independent variable and the binary label pertinent to the task as the dependent variable. Then features with high absolute value of correlation (>c) with other features were removed from the feature set to form a pre-selected feature set. Then a random forest classifier was trained using the pre-selected set of features. The parameters of the random forest classifier (number of features that are selected for each tree and number of cases included in each tree leaf) as well as the parameters *N* and *c* described above were selected through a cross-validation experiment within the training set in which the training and evaluation were repeated multiple times for a range of values of the four parameters. The parameters that provided the highest cross-validation AUC were selected. This procedure was repeated separately for each of the 8 classification tasks resulting in one final model trained using the training set for each of the tasks. Once the parameters were optimised and the models were developed using the training set, they were evaluated on the independent test set using area under the ROC curve metric. Confidence intervals were estimated using the DeLong method^27^ and the significance of the association was established using a logistic regression model using the classifier output as the covariate. A *p*-value <0.00625 (0.05/8 models) was considered significant. An additional analysis was conducted in subgroups formed in the test sets based on the scanner manufacturers (GE and Siemens), race (white and other races), and menopausal status (pre and post). The grouping within the race category was done in order to have a sufficient number of cases in each subgroup (some races have a very low representation in our cohort).

Furthermore, we conducted an exploratory analysis to establish which individual features showed the highest association with the molecular markers. Specifically, for all features, we computed the AUC for the eight prediction tasks on the training set. We sorted the features in descending order according to AUC and removed highly correlated features (|*r*| > 0.5) to select the top (*N* = 10) features for each of the eight prediction tasks. For tasks (1–4), we removed repeated occurrences of any feature to arrive at a set of 37 unique features and calculated the AUCs and confidence intervals for these selected features on the test set. For each of tasks (5–8) we computed AUCs and confidence intervals for 10 features.

All analysis, except for the computation of confidence interval was conducted in MATLAB, 2016b (MathWorks, Natick, Mass). The confidence intervals were computed in R, version 3.4.0 (http://www.r-project.org/).

## Results

Table 2 shows the patient clinical characteristics. The distribution of patients according to molecular biomarkers, in the training and test set shown in Table 3 are very similar.

**Table 2:** Clinicopathological characteristics of the overall patient population, by molecular subtypes, receptor status positivity and Ki-67 availability

Patient characteristics	Entire cohort	Luminal A	Luminal B	HER2	TNBC	ER positive	PR positive	HER2 positive	Ki-67
Number of patients	922 (100%)	595 (64.53%)	104 (11.27%)	59 (6.39%)	164 (17.79%)	686 (74.40%)	598 (64.86%)	163 (17.68%)	450 (48.81%)
Median age (age range) in years	52.25 (21.75–89.49)	53.61 (25.7–89.49)	46.54 (29.78–79.52)	51.92 (27.14–79.08)	50.4 (21.75–80.70)	52.82 (25.7–89.49)	52.42 (25.7–89.49)	48.38 (27.1–79.52)	52.5 (23.98–80.46)
*Race*									
White	651 (70.61%)	442 (74.29%)	75 (72.12%)	36 (61.02%)	98 (59.76%)	510 (74.34%)	453 (75.75%)	111 (68.10%)	332 (73.38%)
Black	203 (22.02%)	107 (17.98%)	21 (20.19%)	15 (25.42%)	60 (36.59%)	123 (17.93%)	103 (17.22%)	36 (22.09%)	88 (19.56%)
Others*	49 (5.31%)	30 (5.04%)	8 (7.69%)	6 (10.17%)	5 (3.05%)	38 (5.54%)	27 (4.52%)	14 (8.59%)	21 (4.67%)
Not available	19 (2.06%)	16 (2.69%)	0	2 (3.39%)	1 (0.61%)	15 (2.19%)	15 (2.51%)	2 (1.23%)	9 (2.00%)
*Menopausal status*									
Pre	407 (44.14%)	240 (40.34%)	59 (56.73%)	23 (38.98%)	85 (51.83%)	293 (42.71%)	263 (43.98%)	82 (50.31%)	198 (44.00%)
Post	499 (54.12%)	344 (57.82%)	43 (41.35%)	36 (61.02%)	76 (46.34%)	380 (55.39%)	323 (54.01%)	79 (48.47%)	249 (55.33%)
Not available	16 (1.74%)	11 (1.85%)	2 (1.92%)	0	3 (1.83%)	13 (1.90%)	12 (2.01%)	2 (1.23%)	3 (0.67%)
*Tumour staging (size)* ^1^									
T1	409 (44.36%)	289 (48.57%)	41 (39.42%)	16 (27.12%)	63 (38.41%)	327 (47.67%)	292 (48.83%)	57 (34.97%)	194 (43.11%)
T2	395 (42.84%)	234 (39.33%)	51 (49.04%)	32 (54.24%)	78 (47.56%)	278 (40.52%)	244 (40.80%)	83 (50.92%)	194 (43.11%)
T3	90 (9.76%)	57 (9.58%)	8 (7.69%)	9 (15.25%)	16 (9.76%)	63 (9.18%)	47 (7.86%)	17 (10.43%)	50 (11.11%)
T4	22 (2.39%)	11 (1.85%)	4 (3.85%)	0	7 (4.27%)	14 (2.04%)	12 (2.01%)	4 (2.45%)	9 (2.00%)
Not available	6 (0.65%)	4 (0.67%)	0	2 (3.39%)	0	4 (0.58%)	3 (0.50%)	2 (1.23%)	3 (0.67%)

ER oestrogen receptor, HER2 human epidermal growth factor, PR progesterone receptor, TNBC triple negative breast cancer. *Includes Asian, Native, Hispanic, Multi, Hawaiian, and American Indian

**Table 3:** Distribution of patients in the training and test sets as per the molecular subtype, receptor status, and Ki-67 values

Molecular marker	Details	Count in training set	Count in test set
Molecular subtype (*N*_TRAIN_ = 461 *N*_TEST_ = 461)	Luminal A	305 (66.16%)	290 (62.91%)
	Luminal B	47 (10.20)	57 (12.36%)
	HER2	27 (5.86%)	32 (6.94%)
	TNBC	82 (17.79%)	82 (17.79%)
ER status (*N*_TRAIN_ = 461 *N*_TEST_ = 461)	Positive	341 (73.97%)	345 (74.84%)
	Negative	120 (26.03%)	116 (25.16%)
PR status (*N*_TRAIN_ = 461 *N*_TEST_ = 461)	Positive	306 (66.38%)	292 (63.34%)
	Negative	155 (33.62%)	169 (36.66%)
HER2 status (*N*_TRAIN_ = 461 *N*_TEST_ = 461)	Positive	74 (16.05%)	89 (19.31%)
	Negative	387 (83.95%)	372 (80.69%)
Ki-67 (*N*_TRAIN_ = 246 *N*_TEST_ = 204)	High	153 (62.20%)	155 (75.98%)
	Low	93 (37.80%)	49 (24.02%)

ER oestrogen receptor, HER2 human epidermal growth factor, PR progesterone receptor

The results from the multivariate models are presented in Table 4. Regarding molecular subtypes, the highest performance was obtained for the models distinguishing Luminal A from other subtypes with AUC = 0.697 (95% CI: 0.647–0.746, *p* < 1.24e−11) and TNBC from the other subtypes AUC = 0.654 (95% CI: 0.589–0.720, *p* < 1.42e−05). The performances for distinguishing HER2 from other subtypes and for Luminal B from other subtypes were somewhat lower and did not reach statistical significance (*p* = 0.03 and *p* = 0.13, respectively). Regarding individual molecular markers, the models showed significant prognostic value for distinguishing ER+ from ER− patients (*p* < 4.2e−06), PR+ from PR− patients (*p* < 1.93e−04). The model for predicting high vs low proliferation (Ki-67) showed AUC = 0.624 with a *p*-value on the margin of significance (*p* = 0.01).

**Table 4:** AUC, CI, and *p*-values obtained for the trained multivariate models in the test set

Name of the Task	AUC in test set with 95% CI	*p*-Value for the model in test set
Luminal A vs other subtypes	0.697 (0.647–0.746)	<0.0001*
Luminal B vs other subtypes	0.566 (0.494–0.638)	0.13
HER2 vs other subtypes	0.633 (0.539–0.727)	0.03
TNBC vs other subtypes	0.654 (0.589–0.720)	<0.0001*
ER positivity vs ER negativity	0.649 (0.591–0.705)	<0.0001*
PR positivity vs PR negativity	0.622 (0.569–0.674)	<0.001*
HER2 positivity vs HER2 negativity	0.500 (0.433–0.567)	0.81
High Ki-67 vs low Ki-67	0.624 (0.531–0.718)	0.01

AUC area under the curve, CI confidence interval, ER oestrogen receptor, HER2 human epidermal growth factor, PR progesterone receptor. *Statistically significant *p* < 0.00625

The results for the subgroup wise analysis using the test set are shown in Table 5. No notable and systematic differences in the performance of the trained models were observed in the subgroups for a majority of the tasks. A minor difference was found in the task of discriminating TNBC patients from other subtypes between the pre and post-menopausal cohorts. Some differences were observed in the discrimination of high Ki-67 from low Ki-67 for different scanner manufacturer, races, and menopausal status.

**Table 5:** AUC and 95% CI obtained for the trained multivariate models in the test set divided into subsets by scanner manufacturer, race, and menopausal status

	Scanner manufacturer		Race		Menopausal status	
	GE (*N* = 284)	Siemens (*N* = 177)	White (*N* = 322)	Other declared races (*N* = 129)	Pre (*N* = 216)	Post (*N* = 235)
Luminal A vs other Subtypes	0.701 (0.638–0.763)	0.685 (0.605–0.765)	0.708 (0.648–0.769)	0.646 (0.55–0.741)	0.652 (0.579–0.725)	0.737 (0.668–0.807)
Luminal B vs other Subtypes	0.579 (0.476–0.683)	0.521 (0.41–0.632)	0.560 (0.467–0.653)	0.543 (0.427–0.659)	0.585 (0.487–0.682)	0.529 (0.417–0.640)
HER2 vs other Subtypes	0.651 (0.534–0.768)	0.625 (0.467–0.783)	0.652 (0.527–0.776)	0.620 (0.472–0.767)	0.612 (0.469–0.548)	0.660 (0.535–0.847)
TNBC vs other Subtypes	0.671 (0.586–0.756)	0.623 (0.52–0.727)	0.658 (0.571–0.746)	0.619 (0.511–0.726)	0.667 (0.582–0.753)	0.612 (0.496–0.727)
ER positivity vs ER negativity	0.638 (0.565–0.712)	0.670 (0.577–0.762)	0.616 (0.542–0.691)	0.669 (0.574–0.765)	0.644 (0.564–0.724)	0.644 (0.559–0.730)
PR positivity vs PR negativity	0.616 (0.549–0.683)	0.633 (0.549–0.717)	0.611 (0.545–0.678)	0.616 (0.516–0.717)	0.627 (0.551–0.702)	0.616 (0.542–0.690)
HER2 positivity vs HER2 negativity	0.500 (0.408–0.593)	0.495 (0.394–0.596)	0.514 (0.428–0.599)	0.505 (0.392–0.617)	0.508 (0.412–0.604)	0.500 (0.405–0.596)
High Ki-67 vs Low Ki-67*	0.648 (0.536–0.760)	0.590 (0.408–0.772)	0.601 (0.495–0.708)	0.725 (0.527–0.922)	0.576 (0.417–0.734)	0.640 (0.520–0.760)

*Number of cases in each category is less than the mentioned counts in second row due to missing Ki-67 values

**Fig. 3:**
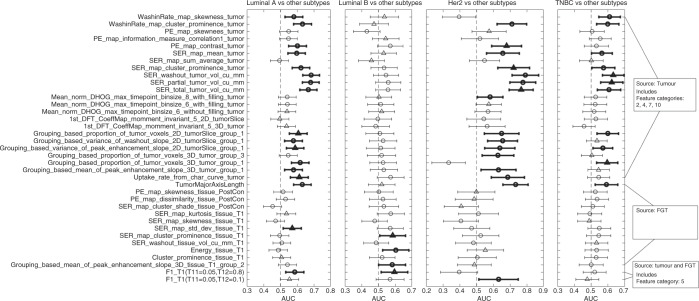
The AUC (indicated by a circular or triangular marker) and confidence intervals (indicated by the endpoints of the lines cutting the marker) values for 37 selected features for prediction tasks 1–4 (indicated at the top of each column). The triangular marker indicates that the corresponding feature was among the 10 features selected from the training set for predicting the corresponding subtype versus others, otherwise the marker is circular. The bold lines indicate that the lower bound of the confidence interval is greater than an AUC of 0.5 and the feature maintained its directionality from the training set

**Fig. 4:**
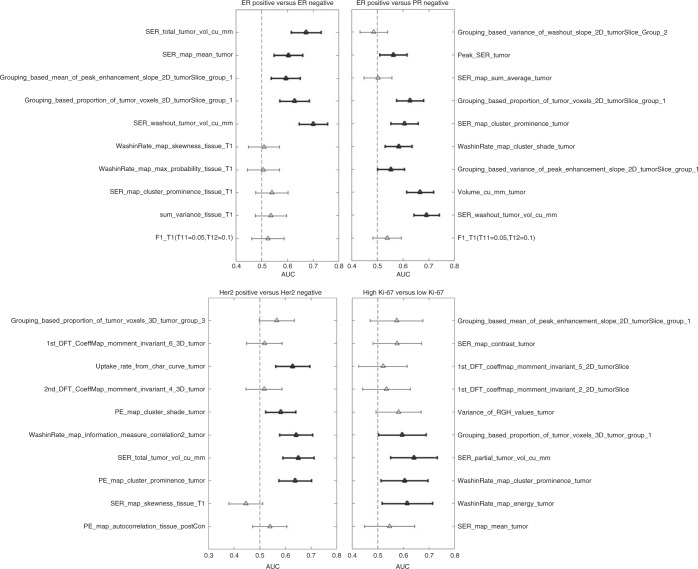
The AUC (indicated by a triangular marker) and confidence intervals (indicated by the endpoints of the lines cutting the marker) values for prediction tasks 5–8 (indicated at the top of each column). The bold lines indicate that the lower bound of the confidence interval is >0.5 and the feature maintained its directionality from the training set

The results for the exploratory univariate analysis are presented in Figs. 3 and 4. The number of features with AUC confidence interval not overlapping with 0.5 was 18, 4, 18, and 11 for the tasks of distinguishing Luminal A, Luminal B, HER2, and TNBC from other subtypes, respectively. All but two of these features maintained their directionality as obtained from the training set. Except for 4 features, all of these features were extracted from the tumour only. Among these 4 features, 2 were extracted from FGT and 2 were extracted using both tumour and tissue, a result consistent with.^15^ The higher values of AUCs were obtained for discriminating HER2 molecular subtype versus others and Luminal A versus other subtypes.

For ER positivity versus ER negativity, 5 features were found to have AUC higher than 0.5 for the lower bound of the confidence interval of AUC. This condition was met by 7, 5, and 4 features for PR positivity versus PR negativity, HER2 positivity versus negativity, and high Ki-67 versus low Ki-67, respectively. All of these features were extracted from tumours.

## Discussion

In this study, we conducted a comprehensive radiogenomic analysis of breast cancer in the context of dynamic contrast enhancement MRI using a machine-learning-based approach. Motivated by the recent surge in the development of new features in breast MRI radiomics and a strong interest in breast cancer radiogenomics, we looked at the effectiveness and generalisability of the features available in the literature as well as features proposed by our group to predict receptor status, proliferation and, surrogate molecular subtypes in a heterogeneous cohort of 922 patients.

Our study demonstrated that there were associations between characteristics of tumours and FGT in dynamic contrast-enhanced MRI and tumour molecular composition. The associations using multivariate models were, however, only of moderate strength with the highest AUC of 0.697 for distinguishing Luminal A from other subtypes. Overall, we demonstrated the strongest associations for OR and PR which suggests that among the imaging features tested, there is differential expression in the imaging phenotype for OR and PR status. Lower levels of association were found for HER2 and Ki-67 which did not reach the level of statistical significance after accounting for multiple hypothesis testing (*p* < 0.03 and *p* < 0.01, respectively). HER2 is a vascular growth factor receptor responsible in part for tumoural angiogenesis. Positive HER2 status has been associated with an increased incidence of multifocal and multicentric disease, increased apparent diffusion coefficient (ADC) scores, and more rapid early enhancement.^28–32^ Higher ADC values have also been associated with lower Ki-67 scores, but diffusion weighted imaging is not typically performed in routine breast MRI and was not included in this study.^33^ If prediction of HER2 and Ki-67 with imaging features is of value, then the inclusion of ADC measurement may be of additional help.

Our results indicate that computer-extracted features might be helpful in identifying biological characteristics of the tumours which help plan patient therapy. However, given the performance of the models, MR imaging features alone could not be used as a non-invasive surrogate of the molecular markers evaluated in this study. The moderate association between MRI features and subtypes demonstrates the promise of such features as part of a composite marker that might include additional clinical variables and imaging features from other modalities to determine tumour genomics. Such a composite biomarker might provide additional, heretofore unknown, benefits to treatment planning that allows for more personalised care. Finally, understanding the relationship between tumour biology and the corresponding radiological phenotype furthers the overall understanding of breast cancer as it informs about specific phenotypical expression of different underlying genomic composition. Our detailed analysis of individual imaging features showed that it is mostly the imaging characteristics of the tumour and less of the normal breast parenchyma that show associations with genomics. However, some features that quantify the relationship between the tumour and normal breast parenchyma enhancement^6^ had radiogenomic associations. Among these tumour features, it was predominantly those that capture enhancement dynamics that showed the highest association with genomics and particularly those related to signal enhancement ratio, which quantifies the relationship between the uptake and the washout times.

A strength of our study is the use of an independent test set which validates that the relationships identified in the training cohort can be generalised to a larger population. The use of a large number of evaluated imaging features and sophisticated machine learning-based multivariate models may result in finding relationships as the result of chance or the result of overfitting the models to the training data. Therefore, the use of a test set to validate the process is of utmost importance. To our knowledge, only two prior investigators have incorporated this important step into their analysis.^3,18^

In this study, we analysed a highly heterogeneous cohort of imaging and patient parameters: (a) age range of women from 21 to 89 years and seven races (b) tumours of all TNM sizes, nuclear grade, OR, PR, and HER2 status (c) 10 different combinations of magnetic field strengths and scanner manufacturers (d) 3 different types of contrast agents were used for the patients (e) range of values applied for image acquisition in terms of slice thickness, repetition times, echo times, acquisition matrices, flip angles and FOVs (e) eight different expert radiologists served as readers. Our additional analysis on subgroups formed using different scanner manufacturers, races, and menopausal status of the patients did not demonstrate major differences in the performance of the trained models the majority of the tasks.

This study had some limitations. While the heterogeneous cohort used in this study allows for more generalisable conclusions, it is likely that that variability in imaging acquisition parameters were a significant source of noise in our analysis and stronger associations could be found in a cohort with uniform imaging parameters. Future analysis could be conducted to evaluate this issue when a larger number of patients scanned in a uniform manner are available.

A future study could also evaluate a variety of image preprocessing techniques that could alleviate the variability in image acquisition. Furthermore, this study relied on surrogate molecular subtypes defined from ER, PR, and HER2 which are not as robustly predictive of outcomes as formal genetic analysis.^34^

In summary, we evaluated associations of imaging variables with the following molecular, genomic, and proliferation characteristics: tumour surrogate molecular subtype, ER, PR, and HER2 status, and the tumour proliferation Ki-67 marker in an independent dataset. We showed moderate associations of imaging features with Luminal A subtype, TNBC, ER, and PR status. This shows a potential for extending the usage of imaging in oncology. However, this needs to be done with caution and likely in conjunction with other variables.

## Electronic supplementary material

Supplemental Materials

## Data Availability

The materials might be made available upon request, some restrictions will apply.
