# Supplementary material for: A machine learning approach to radiogenomics of breast cancer: a study of 922 subjects and 529 DCE-MRI features
Source: Br J Cancer. 2018 Jul 23;119(4):508–16. doi: 10.1038/s41416-018-0185-8 (PMC6134102; doi:10.1038/s41416-018-0185-8)
Supplement: Supplementary file 1 — Supplemental Materials [file 41416_2018_185_MOESM1_ESM.docx]

**Supplementary material for manuscript “A machine learning approach to radiogenomics of breast cancer: a study of 922 subjects and 529 DCE-MRI features”**

**Authors:**

Ashirbani Saha^1^, PhD, Michael R. Harowicz^1^, MD, Lars J. Grimm^1^, MD, Connie E. Kim^1^, MD, Sujata V. Ghate^1^ MD, Ruth Walsh^1^, MD, Maciej A. Mazurowski^1,2,3^, PhD

**Affiliations:**

1. Department of Radiology, Duke University School of Medicine Durham, NC 22705, USA
2. Department of Electrical and Computer Engineering, Duke University, Durham, NC 22705, USA
3. Duke University Medical Physics Program, Durham, NC 22705, USA

Table S1 Patient distribution for different scanner manufacturers and magnetic field strengths

| Characteristics | Magnetic Field Strength | Manufacturer Details | Patient Count |
| --- | --- | --- | --- |
| Magnetic Field Strength | 1.5 T | Optima MR450w, GE Healthcare, Little Chalfont, UK | 98  (10.63%) |
|  |  | Signa HDx, GE Healthcare, Little Chalfont, UK | 53  (5.75%) |
|  |  | Signa HDxt, GE Healthcare, Little Chalfont, UK | 138 (14.97%) |
|  |  | Avanto, Siemens, Munich, Germany | 179 (19.41%) |
|  | 3 T | Signa Excite, GE Healthcare, Little Chalfont, UK | 10 (1.08%) |
|  |  | Signa HDx, GE Healthcare, Little Chalfont, UK | 219 (23.75%) |
|  |  | Signa HDxt, GE Healthcare, Little Chalfont, UK | 110 (11.93%) |
|  |  | Skyra, Siemens Healthcare, Little Chalfont, UK | 57  (6.18%) |
|  |  | Trio, Siemens, Munich, Germany | 1  (0.11%) |
|  |  | Trio Tim, Siemens, Munich, Germany | 57  (6.18%) |

Table S2 Values of Different MRI acquisition parameters in the dataset

| MRI Acquisition Parameters | Value Details | Values |
| --- | --- | --- |
| Slice Thickness (mm) | Range | 1.04 – 2.5 |
|  | Median | 2 |
| Repetition Time (ms) | Range | 3.54-7.39 |
|  | Median | 5.2 |
| Echo Time (ms) | Range | 1.25-2.76 |
|  | Median | 2.39 |
| Acquisition Matrix | Minimum Array Size | 320 × 320 |
|  | Maximum Array Size | 448 × 448 |
| Flip Angle (degrees) | Range | 7-12 |
|  | Median | 10 |
| FOV(mm) | Range | 250-480 |
|  | Median | 350 |
